# Correlation between crystal structure and magnetism in PLD grown epitaxial films of ε-Fe_2_O_3_ on GaN

**DOI:** 10.1080/14686996.2020.1870870

**Published:** 2021-02-04

**Authors:** Sergey M. Suturin, Alexander M. Korovin, Alla A. Sitnikova, Demid A. Kirilenko, Mikhail P. Volkov, Polina A. Dvortsova, Victor A. Ukleev, Masao Tabuchi, Nikolai S. Sokolov

**Affiliations:** a Division of Solid State Physics, Division of Physics of Dielectrics and Semiconductors, Centre of Nanoheterostructure Physics, Ioffe Institute, St. Petersburg, Russia; b Laboratory for Neutron Scattering and Imaging (LNS), Paul Scherrer Institute (PSI), Villigen, Switzerland; c Synchrotron Radiation Research Center, Nagoya University, Nagoya, Japan

**Keywords:** Iron oxides, epsilon ferrite, RT multiferroic, GaN, PLD epitaxial films, reciprocal space mapping, electron and x-ray diffraction, XMCD, 102 Porous / Nanoporous / Nanostructured materials, 202 Dielectrics / Piezoelectrics / Insulators, 203 Magnetics / Spintronics / Superconductors, 212 Surface and interfaces, 306 Thin film / Coatings, 502 Electron spectroscopy, 503 TEM, STEM, SEM, 504 X-ray / Neutron diffraction and scattering

## Abstract

In the present paper we discuss correlations between crystal structure and magnetic properties of epitaxial ε-Fe_2_O_3_ films grown on GaN. The large magnetocrystalline anisotropy and room temperature multiferroic properties of this exotic iron oxide polymorph, make it a perspective material for the development of low power consumption magnetic media storage devices. Extending our recent progress in PLD growth of ε-Fe_2_O_3_ on the surface of technologically important nitride semiconductors, we apply reciprocal space tomography by electron and x-ray diffraction to investigate the break of crystallographic symmetry occurring at the oxide-nitride interface resulting in the appearance of anisotropic crystallographic disorder in the sub-100 nm ε-Fe_2_O_3_ films. The orthorhombic-on-hexagonal nucleation scenario is shown responsible for the development of a peculiar columnar structure observed in ε-Fe_2_O_3_ by means of HRTEM and AFM. The complementary information on the direct and reciprocal space structure of the columnar ε-Fe_2_O_3_ films is obtained by various techniques and correlated to their magnetic properties. The peculiar temperature dependence of magnetization studied by the small-field magnetization derivative method and by neutron diffraction reveals the existence of a magnetic softening below 150 K, similar to the one observed earlier solely in nanoparticles. The magnetization reversal in ε-Fe_2_O_3_ films probed by X-ray magnetic circular dichroism is found different from the behavior of the bulk averaged magnetization measured by conventional magnetometry. The presented results fill the gap between the numerous studies performed on randomly oriented ε-Fe_2_O_3_ nanoparticles and much less frequent investigations of epitaxial epsilon ferrite films with lattice orientation fixed by the substrate.

## Graphical abstract

**Figure:**
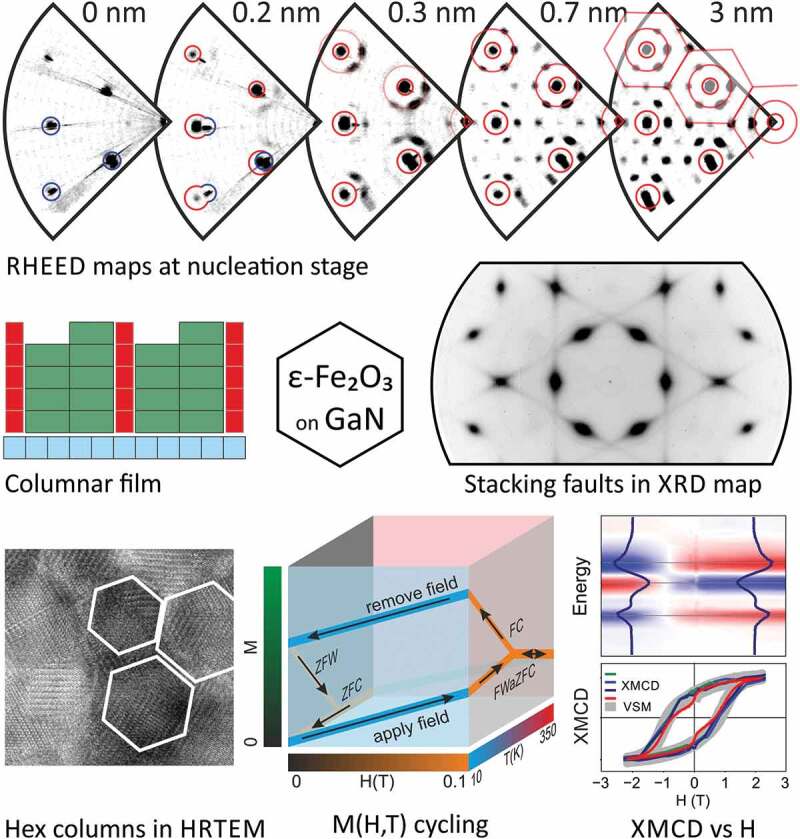

## 1. Introduction

Integration of semiconducting and magnetic materials into a single heterostructure is expected to provide opportunities for designing novel functional spintronic devices. The iron oxides form a vast family of magnetic materials exhibiting a rich variety of outstanding physical properties. Among these the metastable ε-Fe_2_O_3_ is the most intriguing phase not existing in the bulk form. The ε-Fe_2_O_3_ phase is ferrimagnetic with a huge magnetocrystalline anisotropy responsible for the coercivity values usually exceeding 2 T in the nanocrystalline (NC) form [1] and 1 T in epitaxial films [2–4]. In the nanocrystalline form ε-Fe_2_O_3_ exhibits a still not fully explained low-temperature phase transition between 100 and 200 K accompanied by a small decrease of magnetization and a large decrease in the coercivity [5]. High coercive field values, room temperature ferroelectric and magnetoelectric properties [6] makes ε-Fe_2_O_3_ a perspective material to be used in the future low power consumption magnetic media storage devices [7]. The majority of the early works related to ε-Fe_2_O_3_ were dedicated to randomly oriented nanoparticles [7–9]. Fabrication of ε-Fe_2_O_3_ epitaxial layers on STO, Al_2_O_3_ and YSZ [2,6,10,11] was later demonstrated. Quite recently it has been shown [3] that the epitaxial layers of ε-Fe_2_O_3_ (along with various other iron oxide polymorphs) can be controllably grown by pulsed laser deposition on GaN(0001). Coupling a multiferroic material to the semiconducting nitride is supposed to be technologically important for potential extension of the capabilities of the modern AIII-BV based microelectronic devices such as bright LEDs, HEMT transistors for high-power, high-frequency, high-temperature and high radiation resistant applications. Placing a room temperature multiferroic layer with controllable magnetization/polarization in contact with a semiconductor adds the functionality of controlling optical, electronic, and magnetic properties of the heterostructure by applied voltage [12–15]. In our previous papers we have demonstrated by using RHEED and XRD that ε-Fe_2_O_3_ films grow on GaN with the [001] polarization axis perpendicular to the surface and the [100] easy magnetization axis lying in plane [3]. The films of ε-Fe_2_O_3_ were shown to grow on GaN in the same epitaxial process as α-Fe_2_O_3_ and γ-Fe_2_O_3_ polymorphs. Thus a carefully control of the growth parameters (growth temperature above 800°C and oxygen pressure of about 0.2 mbar) is required to drive the growth towards formation of the epsilon ferrite. The ε-Fe_2_O_3_ epitaxial films on GaN were shown to exhibit magnetization reversal loops with a coercive field of up to 2 T and saturation of above 100 emu/cm^3^. An additional soft magnetic component appearing in MH loops can be ascribed to the interface magnetism as followed from the polarized neutron reflectometry studies carried out for ε-Fe_2_O_3_/GaN [4] and ε-Fe_2_O_3_/MgO/GaN [16] films.

The present paper describes a detailed investigation into the growth, crystal structure and magnetic properties of the multiferroic-on-semiconductor epitaxial films of ε-Fe_2_O_3_/GaN. Highlighted are the issues related to the nucleation of the ε-Fe_2_O_3_/GaN interface and the anisotropic disorder appearing at the nucleation stage. The nucleation of this disorder at the oxide-nitride interface and its development into the columnar structure of the thicker epsilon ferrite film is studied by a combination of reciprocal and direct space techniques such as 3D reciprocal space mapping by electron and X-ray diffraction and high-resolution transmission electron microscopy. The relation of the film columnar structure to the magnetic properties of the ε-Fe_2_O_3_ films including the temperature dependence of magnetization is studied by means of bulk averaging vibrating sample magnetometry and iron coordination sensitive X-ray magnetic circular dichroism. For the first time an epitaxial ε-Fe_2_O_3_ film was studied by neutron diffraction in the range of temperatures between 20 K and 300 K. The obtained results are supposed to fill the gap between the numerous studies performed on ε-Fe_2_O_3_ nanoparticles and much less frequent investigations of epsilon ferrite films with lattice orientation fixed by the substrate.

## 2. Materials and methods

The epsilon ferrite films were grown by means of pulsed laser deposition (PLD setup produced by SURFACE, Germany) on the Ga terminated surface of the 3 mkm GaN (0001)/Al_2_O_3_ template fabricated by means of metalorganic vapour-phase epitaxy. The iron oxide was grown from a Fe_2_O_3_ stoichiometric target ablated by CompexPro 201 (Coherent, USA) KrF excimer laser. The growth was performed in oxygen at a pressure of 0.2 mbar with the substrate temperature in the range of 750–850°C. The crystallinity, epitaxial relations and defect structure of the grown layers was monitored by in-situ high energy electron diffraction (RHEED) reciprocal space 3D mapping. Being a substantial improvement over the conventional RHEED [17,18] the reciprocal space tomography deals with a sequence of diffraction patterns taken during the fine-step azimuthal rotation of the sample. The 3D intensity maps built from a series of Ewald spherical sections can be demonstrated in the easily interpreted form of planar cuts or projections along a specific crystallographic axis. The X-ray diffraction studies were carried out on a four-circle diffractometer at BL3A beamline of Photon Factory synchrotron (Tsukuba, Japan). The large volume 3D reciprocal space maps were obtained with a 2D Pilatus 100 K detector in the same way and using the same processing software as described above for RHEED. The in-plane and cross-section transmission electron microscopy studies were carried out at Jeol JEM-2100F (JEOL, Japan) microscope operated in the conventional bright-field and high-resolution imaging modes. The surface morphology was studied using the NTEGRA (NT_MDT, Russia) atomic force microscope operated in the semi-contact mode. Magnetization loops and temperature dependence curves were measured on a PPMS (Quantum Design, USA) vibrational sample magnetometer (VSM) in the temperature range between 10 K and 300 K. The x-ray absorption spectroscopy (XAS) and x-ray magnetic circular dichroism (XMCD) studies have been carried out at RT and 10 K at ID32 beamline of ESRF synchrotron (Grenoble, France) and BL16 beamline of KEK PF synchrotron (Tsukuba, Japan). The measurements were performed in total electron yield (TEY) mode with the magnetic field applied in-plane and the circular polarized photons incident at 30 deg. The neutron diffraction measurements were carried out at the D10 single crystal four-circle diffractometer (ILL, France) [19]. The incoming neutron beam with a wavelength of λ = 2.36 Å was provided by the vertically focusing pyrolytic graphite monochromator. The analyzer option was used to improve the signal over background ratio.

## 3. Results and discussion

### 3.1. Reciprocal lattice evolution at the nucleation stage studied by RHEED

**Figure 1.**
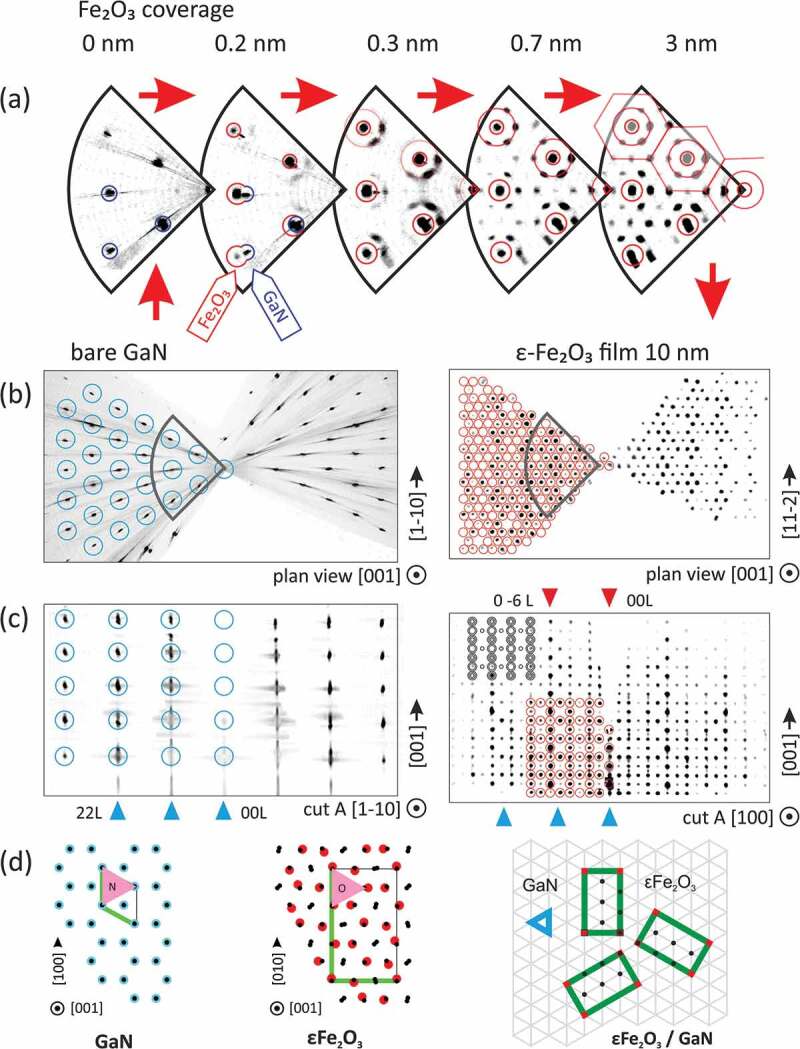
Evolution of reciprocal space structure upon deposition of ε-Fe_2_O_3_ layer on GaN(0001) surface studied by RHEED reciprocal space mapping. Step by step evolution of the in-plane order, showing gradual development of the epsilon ferrite periodicity around the FeO-like rods (a). The in-plane (b) and out-of-plane (c) reciprocal space maps of initial GaN surface (left) and 10 nm thick ε-Fe_2_O_3_ film (right). The in-plane lattice matching between ε-Fe_2_O_3_ (001) and GaN (0001) is schematically shown (d). Part of Fig. 1 is reproduced by permission from [https://doi.org/10.1103/PhysRevMaterials.2.073403], copyright [2018, APS]

The evolution of the crystal structure at the ε-Fe_2_O_3_/GaN interface during the nucleation stage was studied *in situ* by RHEED 3D mapping (reciprocal space tomography). This technique is highly relevant for the study of lateral periodicity as it gives access to the intensity distribution in the reciprocal space plane both perpendicular (out-of-plane diffraction) and parallel to the surface (in-plane diffraction). Figure 1 shows the step-by-step evolution of the reciprocal space structure observed during the growth of a 10 nm thick ε-Fe_2_O_3_ film. Shown are side-view reciprocal space cuts perpendicular to ε-Fe_2_O_3_ [100] and plan-view reciprocal space projections along the ε-Fe_2_O_3_ [001] surface normal. The growth starts on the atomically smooth GaN (0001) surface, the corresponding reciprocal lattice consisting of a hexagonal array of intensity modulated rods stretching perpendicular to the surface (Figure 1(b–c)). Upon Fe_2_O_3_ deposition the hexagonal array of GaN rods gets gradually replaced by a similar (though laterally expanded) hexagonal array of the iron oxide rods. Both arrays can be observed simultaneously at the coverage of 0.2 nm (Figure 1(a)). The 7.5% lateral expansion of the layer pattern corresponds to the in-plane reduction of the anion-to-anion bond lengths from 3.19 Å (N-N) in GaN to 2.91–2.95 Å (O-O) in the 3+ iron oxide [3]. Showing a weak intensity modulation along the rod, the observed pattern corresponds to a flat layer with a 1 × 1 lateral periodicity of about 2.9 Å that only exists in FeO within (111) plane but not in any 3+ iron oxide where the surface cell is considerably larger. E.g. in ε-Fe_2_O_3_ (001) the surface cell is 3 × √3 (5.09 Å × 8.78 Å) as shown schematically in Figure 1(d). Taking into account the geometrical similarity of the anion planes in GaN and Fe_2_O_3_ [3], it is reasonable to assume that ε-Fe_2_O_3_ nucleates in register with GaN. However because of the lower surface symmetry and larger unit cell, the iron oxide islands nucleate out of phase with each other. The only long range order these islands have in common is that of the underlying GaN surface. Therefore the only observed reciprocal space nodes are the nodes that lie close to the nodes of GaN (shown with red circles in Figure 1(a)).

The described 1 × 1 hexagonal structure persists until the iron oxide coverage reaches about 0.3 nm. At this coverage the long range order typical of ε-Fe_2_O_3_ starts to gradually develop as evidenced by the appearance of a circle of 6 neighbor rods around each rod of the fundamental 1 × 1 array (Figure 1(a) 0.3 nm). The neighbor rods appear at exactly where they are expected in ε-Fe_2_O_3_. As more material is deposited, the defects in the underlying atomic layers get healed and the size of crystallographically coherent domains increases. The brightness of the first circle of neighbor rods grows with coverage. Additional rods appear on the circle of a twice larger radius at a coverage of 0.7 nm (Figure 1(a)). At the increased coverage the fully developed ε-Fe_2_O_3_ diffraction pattern appears in both the side and plan views (Figure 1(b–c)) in full agreement with the model (shown with circles). On the [100] zone axis side-view reciprocal space maps the development of the long range order looks as appearing of reflection rows at ±1/6 positions around the 1 × 1 rods (marked with red triangles), and then later at ±2/6 and ±1/2 positions. The observed diffraction patterns are spotty rather than streaky corresponding to the surface consisting of islands, which are in phase with the GaN substrate but not obligatory with each other. The ε-Fe_2_O_3_ [100] axis lies parallel to the one of three equivalent GaN<1-10> directions.

### 3.2. Anisotropic disorder in ε-Fe_2_O_3_ films studied by XRD reciprocal space mapping

**Figure 2.**
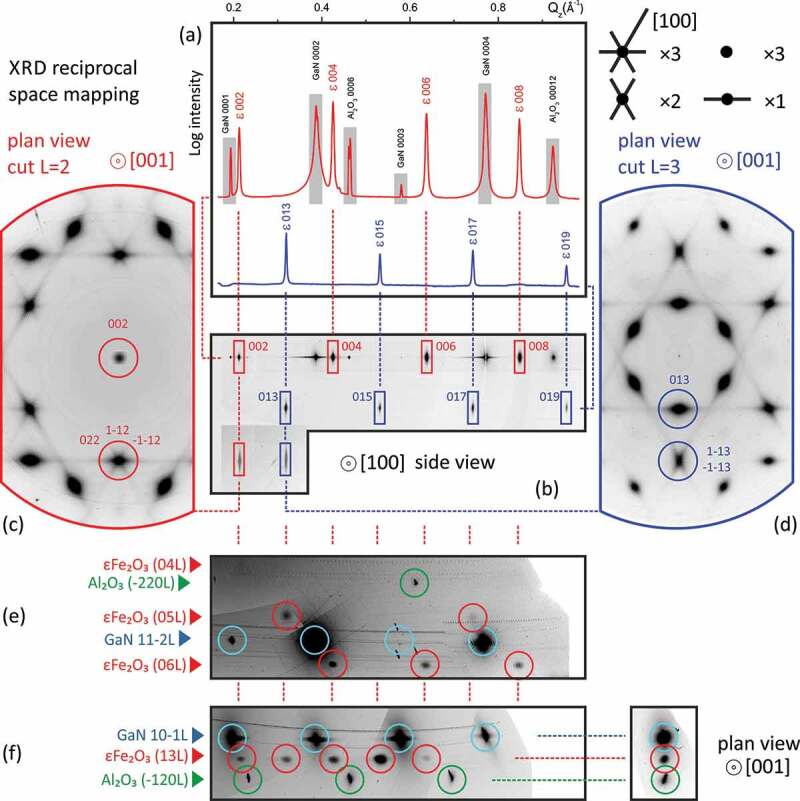
The reciprocal space structure of 80 nm (a-d) and 5 nm (e-f) ε-Fe_2_O_3_ films measured by XRD mapping. Intensity profiles measured along ε-Fe_2_O_3_ [00 L] and [01 L] axes (a). The side-view reciprocal space cut with ε-Fe_2_O_3_ [100] zone axis (b). The plan-view reciprocal space cuts ([001] zone axis) at L = 2 (c) and L = 3 (d) showing the peculiar star-like shape of ε-Fe_2_O_3_ reflections. Reciprocal space cuts in the vicinity of the GaN rods (e, f) show that only reflections with GaN-like in-plane periodicity are present in the thin iron oxide film

While RHEED is indispensible for the *in situ* studies of the crystal structure at the film surface, the bulk-averaged data is more reliably obtained by X-ray diffraction. The results of XRD reciprocal space mapping studies carried out for a 80 nm thick ε-Fe_2_O_3_ film are presented in Figure 2. The wide area reciprocal space cut in Figure 2(b) shows a series of ε-Fe_2_O_3_ 00(2 n), Al_2_O_3_ 000(6 n) and GaN 000(2 n) specular reflections along with a row of the off-specular ε-Fe_2_O_3_ (0, 1, 2 n + 1) reflections. The intensity profiles drawn along 00 L and 01 L are shown in Figure 2(a). Absence of the intensity maximums corresponding to the other iron oxide polymorphs such as γ-Fe_2_O_3_ [16] or α-Fe_2_O_3_ [3] confirms the crystallographic purity of the grown epsilon ferrite and allows estimation of the lattice parameters as follows: a = 5.09 Å, b = 8.78 Å, c = 9.44 Å.

Remarkably the off-specular reflections in thick ε-Fe_2_O_3_ films exhibit a peculiar star-like shape as seen on the L = 2 (Figure 2(c)) and L = 3 (Figure 2(d)) reciprocal space cuts carried out parallel to the film surface. The number of streaks in the star-like reflection depends on how many crystallographic domains contribute to it. This follows from the epitaxial relations observed in the orthorhombic-on-hexagonal ε-Fe_2_O_3_/GaN system [3]: GaN[1–10] || ε-Fe_2_O_3_ [100] or [−1-10] or [−110]. With this three-fold ambiguity there exist three equivalent ε-Fe_2_O_3_ crystallographic domains rotated around the c-axis by 120 deg with respect to each other. With *b* lattice parameter in ε-Fe_2_O_3_ being √3 times larger than *a*, a great deal of reciprocal space nodes coincide upon ±120 deg lattice rotation around the surface normal. The nodes coincident for all three domains produce 3-streak stars while the 2-streak stars appear around the nodes belonging to just two domains. The single domain nodes showing a single streak are the most informative as they allow identification of the elongation direction as ε-Fe_2_O_3_ [100], which is by coincidence the easy magnetization axis in ε-Fe_2_O_3_.

Thus we conclude that the observed complex diffraction pattern in ε-Fe_2_O_3_/GaN films is a combination of three equivalent patterns at 120 deg to each other with reflections in each pattern being elongated uniaxially. Such uniaxial elongation can be often attributed to the break of the long range order in the streak direction, e.g. perpendicular to a twin or an antiphase boundary [18,20] or more generally to the lack of a well-defined periodicity in the particular direction [21]. Interestingly the triple-coincident reflections located close to the GaN rods are laterally narrow, have round shape and show no streaks (see the 002 reflection in Figure 2(c) and the row of 13 L off-specular reflections in Figure 2(f)). The fact that the GaN-like periodicity is not disturbed by the defects is in agreement with the RHEED data showing that the earliest and the best developed long range order in a thin ε-Fe_2_O_3_/GaN film is related to the periodicity of the host gallium nitride surface. The early development of the GaN-like order is also evident from the XRD maps (Figure 2(e–f)) measured in a 2 nm ε-Fe_2_O_3_ film. The bright ε-Fe_2_O_3_-like reflections are only present in the vicinity of GaN 11–2 L and 10–1 L reciprocal space rods and missing elsewhere. Interestingly, very similar XRD maps were observed at the early stage of the iron oxide films growth at lower temperature of 600°C and oxygen pressure of 0.02 mbar. Though these growth conditions are known [3] to result in development of α-Fe_2_O_3_ phase, we conclude that the few nm thick transition layer at the interface in these films has an epsilon-like rather than alpha-like crystal structure. The same reciprocal space structure was also observed in the few nm Fe_2_O_3_ film grown at a much lower temperature of 400°C and then annealed to 800°C.

To summarize the previous sections, a specific anisotropic disorder (which is a variable range order dependent on the reciprocal space vector length and direction) was observed in ε-Fe_2_O_3_/GaN system by RHEED and XRD reciprocal space tomography techniques. The disorder presumably appears at the early iron oxide growth stage in a wide range of growth conditions and is due to the symmetry break at the nitride-oxide interface. The disorder then propagates into thicker ε-Fe_2_O_3_ films as more material is deposited. In the next section this phenomenon will be discussed from the point of view of transmission electron microscopy – the method operating in direct rather than reciprocal space.

### 3.3. The columnar structure of thick ε-Fe_2_O_3_ films studied by TEM

**Figure 3.**
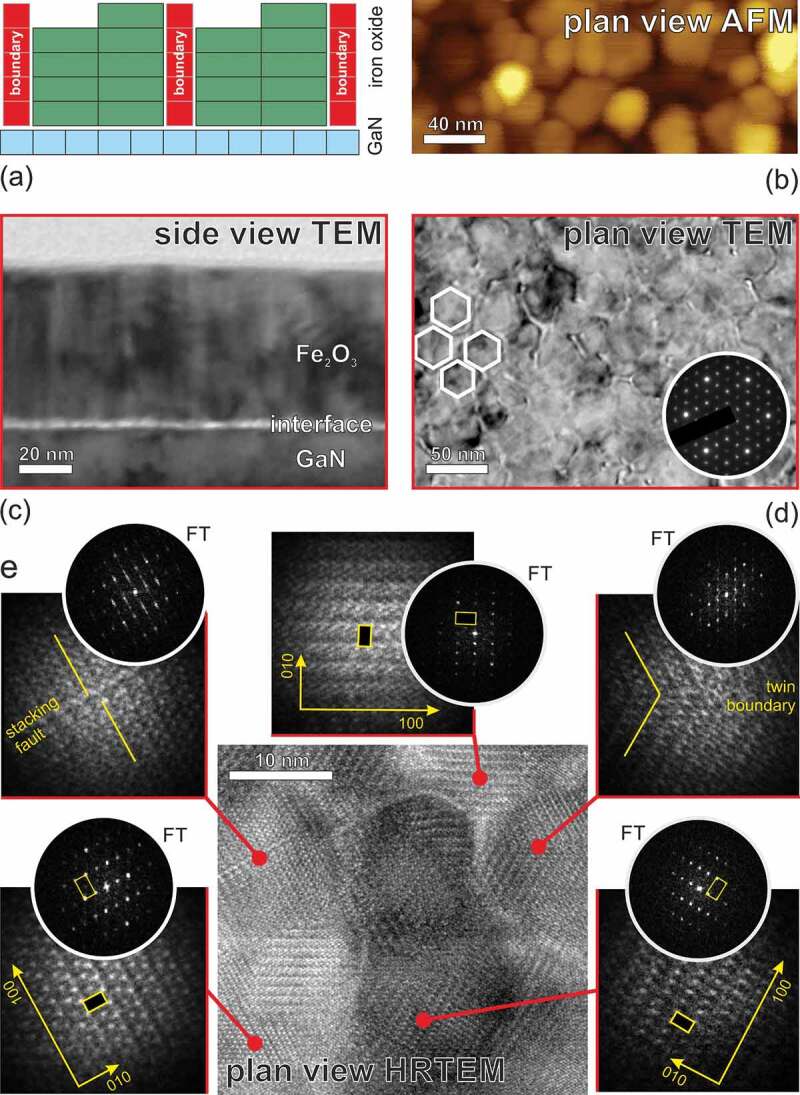
Schematic presentation of the columnar film structure (a) in agreement with the AFM image (b). Cross section TEM image (c) showing the main film and the transition layer. Typical in plane TEM (d) and HRTEM (e) images showing the columnar structure of the ε-Fe_2_O_3_/GaN film. The insets show a magnified view of the atomic structure within columns and the corresponding Fourier transforms. The stacking faults and twin boundaries seen in some columns are responsible for the reciprocal space node elongation in the [100] direction

The cross-section TEM image of a 60 nm ε-Fe_2_O_3_ film presented in Figure 3(c) suggests that the film has a columnar structure, consisting of vertical flat topped columns of approximately 15–25 nm in diameter. As mentioned in out earlier studies [4], TEM is able to recognize the few nm thick transition layer at the Fe_2_O_3_/GaN interface due to its lower density and inferior crystallographic order as compared to the main iron oxide film. The transition layer looks rich of defects that accommodate the lattice difference between the film and the substrate. The lack of a well-defined crystal structure is in agreement with the early stage RHEED maps showing no features of the orthorhombic ε-Fe_2_O_3_ lattice.

The in-plane morphology studies of the thick ε-Fe_2_O_3_ films have been performed by AFM and TEM. The AFM image (Figure 3(b)) shows a dense array of mounds of approximately 30 nm in diameter. A better resolved TEM image (Figure 3(b)) reveals that these mounds have hexagonal shape presumably corresponding to the tightly packed flat topped ε-Fe_2_O_3_ columns. The high resolution in-plane TEM image presented in Figure 3(e) shows in more detail the atomic structure within each ε-Fe_2_O_3_ column. In agreement with the RHEED and XRD data presented above, the atomic rows are oriented in the three possible directions at 120 deg to each other. The observed unit cell has an approximate size of 5 Å × 8.7 Å which corresponds well to the *a-b* dimensions of the orthorhombic ε-Fe_2_O_3_ unit cell. The Fourier transforms shown alongside the zoomed regions are in agreement with the ε-Fe_2_O_3_ reciprocal lattice viewed along the [001] axis. For the different iron oxides such as α-Fe_2_O_3_ and γ-Fe_2_O_3_ the reciprocal lattice nodes viewed from this direction would form a regular hexagonal lattice. Remarkably, the same hexagonal lattice results if the Fourier transform is applied to the large area TEM image or if the large area electron diffraction pattern is measured (Figure 3(d)).

The boundaries between the columns contain complex defects appearing upon coalescence of the adjacent nucleation grains. It must be noted that there is a great number of equivalent ways of placing the large surface cell of ε-Fe_2_O_3_ (001) over the short period surface of GaN (Figure 1(d)). Therefore the defects between the columns are more complex than just being the ABCCBA stacking faults. The adjacent columns are practically incoherent to each other. The Fourier transform of the area containing multiple columns looks as a simple superposition of the reciprocal space nodes of each domain. More regular defects like twin boundaries and stacking faults are observed within the same hexagonal column. These can be caused by the growth perturbations. In contrary, the regions on both sides of the antiphase or twin boundary within the same column are highly coherent, which results in the reciprocal space node elongation in the [100] direction. These elongations are apparently of the same nature as the XRD streaks described earlier (Figure 2). The observed HRTEM images resemble those presented in Ref [22]. where a 200 nm Ga_2_O_3_ film was grown by means of MOVPE onto the Al_2_O_3_ substrate. Similar to our study the Ga_2_O_3_ crystal structure was identified as orthorhombic Pna_21_ with the c-axis perpendicular to the surface. It was claimed by the authors that only the ABAC stacking in the cation sublattice is subject to twinning while the slightly distorted close packed lattice of oxygen atoms remains unchanged.

### 3.4. Magnetization reversal in ε-Fe_2_O_3_ films of different thickness

**Figure 4.**
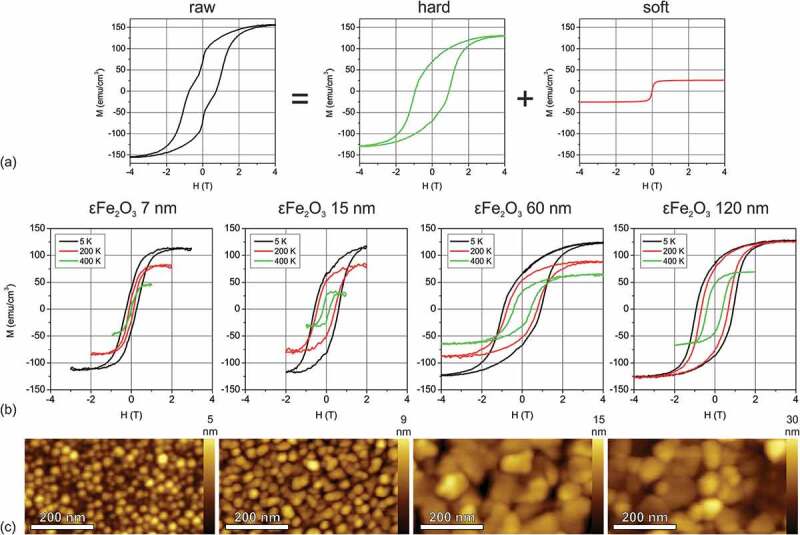
The decomposition of the typical wasp-waist MH loop in ε-Fe_2_O_3_ into hard and soft magnetic components (a). Magnetization curves measured in ε-Fe_2_O_3_ films of different thickness at 5 K, 200 K and 400 K by VSM (b). The temperature independent soft magnetic component is subtracted for easier comparison. The columnar morphology of the same series of films measured by AFM (c)

The field and temperature dependence of magnetization in ε-Fe_2_O_3_/GaN films have been studied in the present work using the conventional vibrating sample magnetometer (VSM) and in more detail by a synchrotron method of X-ray magnetic circular dichroism (XMCD). Figure 4(a) shows a typical MH curve observed in a 60 nm thick ε-Fe_2_O_3_ film at T = 5 K. As was noticed in the earlier works [2–4,6-11] the magnetization loops in ε-Fe_2_O_3_ usually show a characteristic wasp-waist shape with an abrupt magnetization jump at H = 0, corresponding to the coexistence of magnetically hard and soft components. The wasp-waist magnetization loop in Figure 4(a) can be qualitatively decomposed into the hard and soft magnetic components by subtracting a temperature independent phenomenological arctangent M = 2⋅M_soft_/π⋅arctan(H/H_soft_) with the parameters of M_soft_ = 10–30 emu/cm^3^ and H_soft_ = 65–75 mT chosen to eliminate the magnetization jump at H = 0. The magnetic properties of the ε-Fe_2_O_3_ films depend drastically on the film thickness as presented in Figure 4(b) showing the magnetization curves measured at T = 5 K, 200 K and 400 K in 7–120 nm thick ε-Fe_2_O_3_ films. For easier comparison the temperature independent soft magnetic component contribution has been subtracted. The value of the low temperature M_s_ is mostly the same for different thickness and is slightly above 100 emu/cm^3^ being a reasonable value for ε-Fe_2_O_3_ [7,23,24]. A drastic decrease of saturation magnetization M_s_ with temperature was observed in all the films. Remarkably the coercive field is shown to increase with the film thickness. A similar size dependence of the coercivity was observed earlier in the ε-Fe_2_O_3_ nanoparticles [7] and was attributed to the superparamagnetic behavior of the small particles (size threshold estimated as 7.5 nm). The coercivity increase was observed up to the particle diameter of about 20–30 nm. Unlike the samples with randomly oriented nanoparticles, the ε-Fe_2_O_3_/GaN films consist of crystallographically oriented columns as shown by TEM. As seen from the series of AFM images shown in Figure 4(c), the column diameter exhibits a synchronous increase with the film thickness which can naturally lead to the increase of coercivity following the same reasons as described for the nanoparticles.

**Figure 5.**
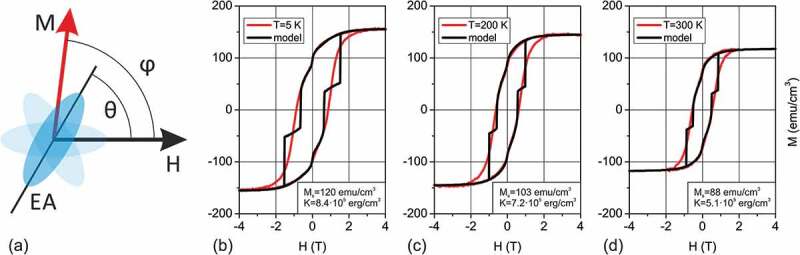
Tentative modeling of the in-plane M(H) curves in a columnar ε-Fe_2_O_3_ film carried out using the Stoner-Walfharth model. Schematic representation explaining the mutual orientation of the magnetic field and the easy axis (a). Magnetization curves measured at 5 K (b), 200 K (c) and 300 K (d) for a 80 nm ε-Fe_2_O_3_ film and the corresponding modeling

The shape of the in-plane VSM magnetization curves has been described in the present work following the Stoner Wolhfarth (SW) approach [25] of weakly interacting magnetic particles, which is the simplest way to describe magnetization reversal in the systems consisting of nanoscale objects that are small enough to contain single magnetic domains [26,27]. The columns in ε-Fe_2_O_3_ film can be considered as such weakly interacting objects as they are crystallographically non-coherent to the neighbors, have differently oriented easy magnetization axes at 120 deg to each other and are small enough to be single domain. To fit the experimentally observed in-plane magnetization curves, the SW model was applied to model the hard magnetic component while the soft magnetic component (giving the characteristic wasp-waist look to the MH loop) was phenomenologically modeled as a temperature independent M_0_⋅tanh(H/H_0_) with M_0_ = 10 emu/cm^3^ and H_0_ = 0.07 T. The results of modeling are shown in Figure 5 for a 80 nm thick ε-Fe_2_O_3_ film. A reasonably good fitting was obtained for the MH loop regions in which magnetization decreases from saturation (H = +4 T) to zero (H = -H_c_). This branch of the loop corresponds to the reversible rotation of M vectors in all three 120 deg domains. At the point where magnetization gets close to zero, the SW model predicts sudden flips of the M vector resulting in abrupt magnetization jumps. The much smoother magnetization reversal observed in the experiment may be due to the collective magnetic behavior of the columns, pinning to the defects or to the size distribution that results in widening of the magnetization flip region. It must be noted that the non-rectangular shape of the magnetization loop with a decreased H_c_ follows from the fact that the three domains are magnetized at the same time. Only one easy axis is aligned parallel to the field while the other two are oriented at 120 deg to the each other and the field. From this point of view, fabricating a film with the true single axis anisotropy is a challenge that might lead to a better understanding of the magnetization dynamics in this material.

### 3.5. Magnetic softening in in ε-Fe_2_O_3_ at 100-150 K

**Figure 6.**
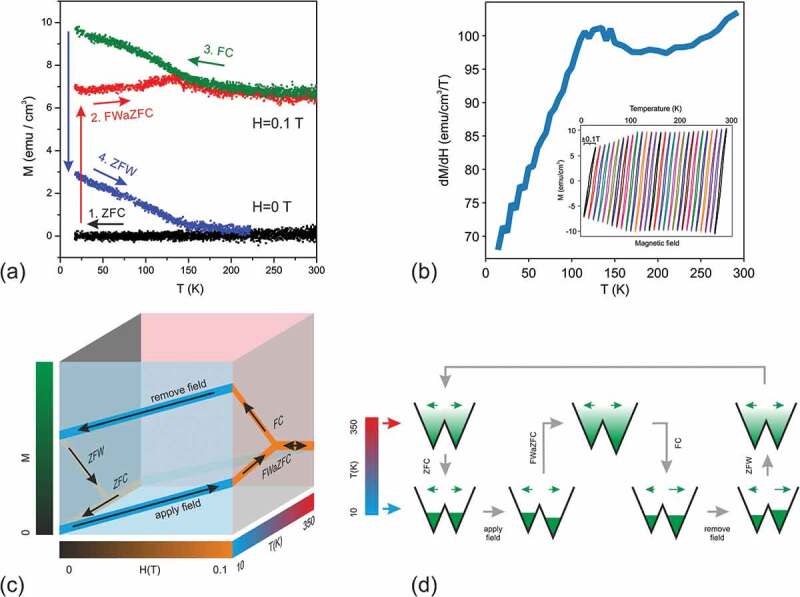
Temperature dependence of magnetization in a 80 nm ε-Fe_2_O_3_ film measured along the ZFC-FWaZFC-FC-ZFW cycle (a). The field-temperature cycle is shown schematically in panel (c). The redistribution of magnetic moments parallel and antiparallel to the field that occur during cycling is shown in panel (d). The small-field dM/dH derivative temperature dependence shows mild magnetic softening in ε-Fe_2_O_3_ in the range of 100–150 K (b). Inset in panel (d) shows MH loops used to calculate the dM/dH derivative. The loops measured in a ± 0.1 T field range are displayed shifted horizontally for better visibility

More information on the properties of the nano-scale magnetic materials can be extracted from the temperature behavior of the magnetization measured along the field-cooling-zero-field-cooling (FC-ZFC) scenario [28–30] consisting of consecutive warming/cooling of the initially demagnetized sample in a small magnetic field. In the present work the classic FC-ZFC scenario has been expanded to a full MT cycle consisting of two constant-field temperature dependences intermediated with the two constant temperature field dependencies (see schematic representation in Figure 6(a)). The result of such measurements performed in a 120 nm ε-Fe_2_O_3_/GaN film are shown in Figure 6(a). The initially demagnetized sample (equal number of magnetic moments along both directions of the easy axis) was first zero field cooled (ZFC) to T = 10 K. A small field of 0.1 T was then applied to reversibly magnetize the sample. At this point the number of parallel/antiparallel magnetic moments is still the same, the non-zero net magnetic moment achieved through reversible rotation. During the subsequent field-warming-after-ZFC (FWaZFC) branch the sample temperature was brought to 300 K. The observed gradual increase of magnetization along this branch is usually explained by the onset of the non-reversible magnetization switching assisted with the thermal motion of magnetic moments (and magnetic softening of ε-Fe_2_O_3_ as will be shown later). On the subsequent field cooling (FC) branch (cooling to T = 10 K) the magnetization followed the same path down to about T = 150 K at which point the FWaZFC and FC curves split drastically apart visualizing the accumulation of the net magnetic moment being most intensive between 50 K and 150 K. The amount of magnetization accumulated during the FWaZFC-FC cycling can be estimated once the field at 10 K is switched off and the magnetization returns to M = 4.5 emu/cm^3^ instead of zero. To complete the field-temperature cycle, the sample was zero-field-warmed (ZFW) up to T = 300 K and then zero-field-cooled (ZFC) back to T = 10 K. The ZFW-ZFC branches run below and almost parallel to the FWaZFC-FC branches described above (Figure 6(a)) returning the magnetization back to zero. It may be concluded that the magnetization in ε-Fe_2_O_3_ being brought out of equilibrium by applying field to a non-magnetized sample (or removing field from a magnetized sample) may be effectively brought to the equilibrium by cycling temperature between 10 K and 150 K (similar to a more traditional field cycling).

In works dealing with magnetic nanoparticles, the M(T) curve splitting is often ascribed to the superparamagnetism [7,28–30]. The superparamagnetic limit for the randomly oriented ε-Fe_2_O_3_ nanoparticles was estimated as 7.5 nm in Ref [7]. This is considerably smaller than the column size in the studied ε-Fe_2_O_3_/GaN films. With magnetocrystalline anisotropy in ε-Fe_2_O_3_ of K = 5–8⋅ 10^5^ erg/cm^3^ (estimated in the previous section) and the column volume of V = 2⋅10^4^ nm^3^ (from AFM, TEM studies), the blocking temperature would be above T_B_ = K⋅V/25 k_B_ = 2900 K, far above the splitting temperature of 150 K observed in M(T) experiment. This rules out the simple superparamagnetic explanation of the observed M(T) curve behavior. To make possible the FWaZFC-FC curve splitting through temperature assisted magnetization switching at 150 K, the magnetocrystalline anisotropy should drop by about one order of magnitude in the vicinity of the splitting temperature. Such a drop was indeed reported to occur in ε-Fe_2_O_3_ nanoparticles [1,5,31] where a large decrease in the coercivity (from 2.25 to 0.08 T) was observed between 100 and 200 K. Surprisingly the same effect has been never explicitly observed in the epsilon ferrite epitaxial films [3,4] including those grown on GaN [3,16].

In the present work we have succeeded in detecting the similar magnetic softening in ε-Fe_2_O_3_ films by using an alternative way to probe the temperature dependence of the magnetic susceptibility. The small field dM/dH derivative has been measured in a demagnetized ε-Fe_2_O_3_ sample by measuring a sequence of small-span M(H) loops during the warming 10–300 K and cooling 300–10 K branches (see inset in Figure 6(b) where the loops are shown with a horizontal offset for better visibility). The field span of 0.1 T was chosen sufficiently small to make the loops fully reversible (magnetization rotation with no switching). The temperature dependence of the loop amplitude plotted in Figure 6(b) reflects the behavior of the magnetic susceptibility. A noticeable peak in dM/dH at around 110 K fits well to the idea of magnetic softening of ε-Fe_2_O_3_ at around this temperature. The coercivity quenching in the ε-Fe_2_O_3_ epitaxial films might be not as temperature-sharp as in the nanoparticles due to a wider size distribution of the columns inside the films. During the temperature ramp, the ‘magnetic softening’ (and the corresponding speed up of the thermo activated switching) proceeds column-by-column being spread across a wider temperature range. The M(T) curves presented in Figure 6(a) were acquired at the temperature ramp rate of about 6 K/min. For the few times lower ramp rates the branches above 150 K tended to separate. This looks reasonable as the switching rate is higher when the temperature is ramped slower. The suggested derivative method allows distinguishing the temperature behavior of susceptibility (reversible) from the temperature activated switching (irreversible). Indeed there is a drastic difference between the FWaZFC-FC pair and the ZFW-ZFC pair. The first one is taken at H = 0.1 T and reflects both temperature changes in dM/dH (see the bump at 110 K) and the switching rate. The latter is taken at zero field and shows no bump at 110 K reflecting just the switching rate.

### 3.6. Neutron diffraction

**Figure 7.**
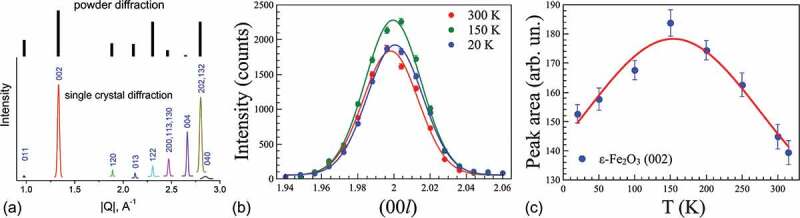
Neutron diffraction reflection intensities observed in a 120 nm ε-Fe_2_O_3_/GaN film compared to the neutron powder diffraction experiment on ε-Fe_2_O_3_ nanoparticles [5] (a). The (002) diffraction peak measured in ε-Fe_2_O_3_ film at 300 K, 150 K and 20 K. Solid lines correspond to Gaussian fits. (b) The temperature dependence of the (002) peak area extracted from the fit (c)

The neutron diffraction measurements were carried out in ε-Fe_2_O_3_/GaN film to investigate peculiarities of magnetic structure in epsilon ferrite. Several specular and off-specular ε-Fe_2_O_3_ reflections could be measured despite the quite low diffraction intensity observed in a 120 nm thick film with an area of 10 × 10 mm^2^. Figure 7(a) shows the comparison between our results and those observed earlier [5] by neutron powder diffraction in ε-Fe_2_O_3_ nanoparticles with the average size of 19 nm. Despite the same symmetries (space group #230 Pna_21_) and lattice constant values, the intensities of the peaks differ significantly, presumably, due to the difference in the oxygen atomic positions in the unit cell. The statistics and the number of the measured peaks did not allow a refinement of the crystal and magnetic structures. Despite the low signal over background ratio it was possible to track down the temperature-dependence of the most intense ε-Fe_2_O_3_ (002) peak (Figure 7(b)). The (002) peak area determined from the Gaussian fit of the (00 *l*) scans shows non-monotonous temperature dependence with a maximum at *T* ≈ 150 K (Figure 7(c)), being in correlation with the magnetization trends measured in similar films by VSM. The neutron diffraction experiment thus directly shows that the M(T) trend is *intrinsically* related to the magnetic structure of ε-Fe_2_O_3_ and not to the other iron oxide polymorphs or impurities that might be present in the film. In contrast to the εFe_2_O_3_ nanoparticles [5], no signature of the incommensurate magnetic satellites is observed in the film. The intensity change of the (002) peak with temperature can be ascribed to the re-orientation of the magnetic moment from the (ab)-plane towards the c-axis, or an antiferromagnetic canting of the spins in the (ab)-plane. Assuming the strong shape anisotropy in the epsilon ferrite film, the second scenario is more plausible. A more detailed investigation is required to draw a conclusion. Such study will be possible with thicker ε-Fe_2_O_3_ films allowing the measurement of the less intense peaks.

### 3.7. X-ray absorption and magnetic circular dichroism studies

**Figure 8.**
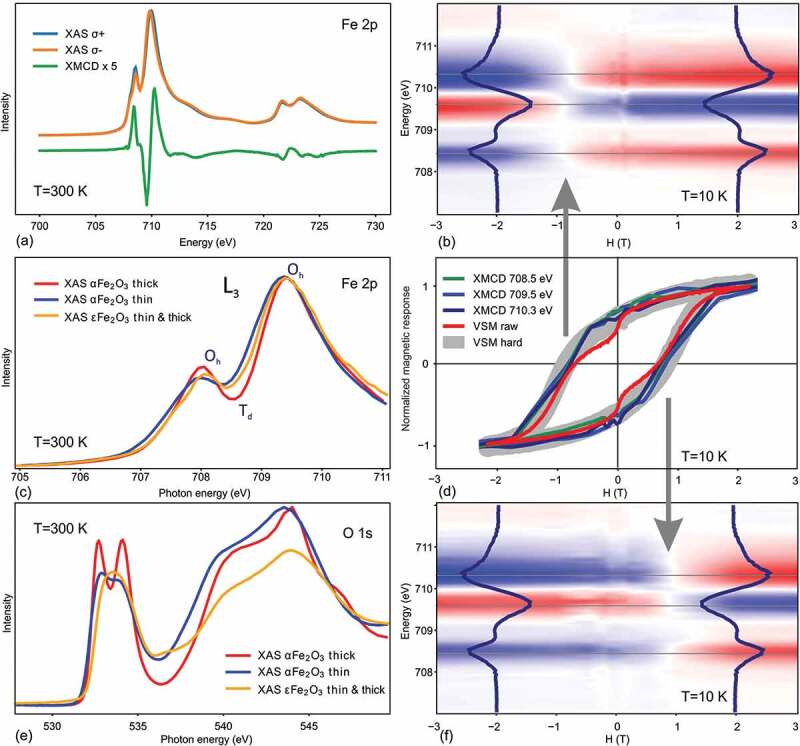
XAS and XMCD spectra of thick ε-Fe_2_O_3_ film measured at Fe L_23_ edge (a). XAS spectra at Fe L_23_ (c) and O K (e) edges in ε-Fe_2_O_3_ compared to those measured in thin and thick α-Fe_2_O_3_ layers. XMCD shape evolution during magnetization reversal along forward (b) and backward (f) field span. Comparison of magnetization curve shapes measured by XMCD at various energies and by VSM (d). The VSM curve is shown as measured (red) and with magnetically soft component subtracted (gray). The loops are normalized vertically for easier shape comparison

X-ray absorption spectroscopy (XAS) and X-ray magnetic circular dichroism (XMCD) have been applied in this work to study chemical environment and magnetic state of the iron atoms in Fe_2_O_3_/GaN films of different thickness. A typical Fe L_23_ absorption spectrum of ε-Fe_2_O_3_ shown in Figure 8(a) (and in more detail in Figure 8(c) is shaped very similar to that of γ-Fe_2_O_3_ [3] as Fe^3+^ ions in both polymorphs reside in octahedral and tetrahedral coordination. For iron in pure octahedral coordination (like in thick α-Fe_2_O_3_/GaN [3]) the absorption looks quite different [31–33] showing a deep intensity depression at 708.5 eV (Figure 8(c)). Interestingly this depression is missing in the few nm transition layer preceding the nucleation of α-Fe_2_O on GaN [3], the corresponding Fe L_3_ edge shape being more ε-Fe_2_O_3_–like rather than α-Fe_2_O_3_–like (Figure 8(c)). A similar trend is observed in the XAS spectra measured at the K-edge of oxygen (Figure 8(e)). The absorption observed in a thin pre-α-Fe_2_O_3_ film looks very similar to ε-Fe_2_O_3_ (a broad peak at 533 eV) and different from the typical absorption of bulk-like α-Fe_2_O_3_ (two split peaks at 532.7 eV and 534.1 eV) [34,35]. The XAS data is in good agreement with the XRD results presented above, confirming that the iron oxide transition layer tends to nucleate in epsilon-like orthorhombic structure even when the growth conditions are not optimized for it.

The X-ray magnetic circular dichroism at L_23_ edge of the transition elements measured in TEY mode is known to provide valuable information about the near-surface nanoscale magnetism [31,36,37]. A typical XMCD spectrum of ε-Fe_2_O_3_/GaN film measured in saturation (H = 3 T) is presented in Figure 8(a). The dichroic signal is relatively weak due to a rather small magnetic moment of about 100 emu/cm^3^ in ε-Fe_2_O_3_. Like in the other iron oxides, the XMCD spectrum features three peaks [31,32] of which the two negative peaks correspond to the Fe in O_h_ coordination (M parallel to H) and the positive peak corresponds to the Fe in T_d_ coordination (M antiparallel to H) [38–43]. To track the magnetic response of individual iron sublattices we have measured XMCD spectra across the iron L_3_ edge spanning the magnetic field back and forth between −4 T and +4 T. The measurements were performed at T = 10 K to maximize magnetic response of epsilon ferrite. The typical field-energy maps obtained in a 60 nm ε-Fe_2_O_3_ film are shown in Figure 8(b,f). Because of the magnetic hysteresis, the forward scan map in Figure 8(b) exhibits an about 2 T offset with respect to the backward scan map in Figure 8(f). The constant energy cross-sections through the maps provide energy dependent magnetization curves. Most informative are the cross-sections through the peaks located at E = 708.5 eV, 709.5 eV and 710.3 eV. Representing contributions from differently oriented magnetic moments residing at one T_d_ and three O_h_ sublattices, the corresponding XMCD-vs-field loops show different amplitudes and signs. To facilitate shape comparison, the loops shown in Figure 8(d) have been normalized to same intensity span. Within experimental accuracy, the shown XMCD loops do not show the characteristic kink at H = 0 that is always present in VSM data. With the kink in the form of a phenomenological arctangent being subtracted from the VSM curve, all the loops become of almost identical shape when normalized vertically (Figure 8(d)). The absence of the kink in the surface sensitive XMCD makes us conclude that the magnetically soft component responsible for the kink is located deep below the surface at Fe_2_O_3_/GaN interface [4] where it can be only sensed by the bulk averaging VSM method. Upon careful analysis we conclude that the shapes of the normalized XMCD field curves measured at different energies are very similar to each other. This confirms that as the external magnetic field is changed, the four Fe sublattices in ε-Fe_2_O_3_ behave synchronously although with different strength and sign. This is at least true in the near surface region of the film probed by TEY. To investigate deeper regions (e.g. the most interesting region at the ε-Fe_2_O_3_/GaN interface), a more depth sensitive technique such as X-ray magnetic resonance reflectometry is supposed to be helpful [44].

## 4. Conclusions

To summarize, we present a detailed investigation into the growth, crystal structure and magnetic properties of the multiferroic-on-semiconductor epitaxial films of ε-Fe_2_O_3_/GaN. The reciprocal space mapping techniques utilizing diffraction of electrons and X-rays were used in this paper to reveal a peculiar anisotropic disorder developed during the nucleation of the orthorhombic ε-Fe_2_O_3_ on the hexagonal GaN. The observed anisotropic disorder is characterized by variable range order dependent on the reciprocal space vector length and direction. The fingerprint of the anisotropic disorder is present in the reciprocal space maps in the form of the star-like reflection widening with the number of star streaks dependent on the reflection multiplicity. This effect is observed in a wide range of growth conditions and is due to the symmetry break at the nitride-oxide interface. The lattice order and periodicity inherited from the underlying GaN surface is found to be unaffected as the independently nucleated iron oxide islands grow in phase with the substrate. The propagation of the anisotropic disorder into the growing ε-Fe_2_O_3_ film results in the development of a columnar structure clearly visible in TEM and AFM images. The internal crystal structure obtained from the high resolution in-plane TEM images is in agreement with the existence of the three equivalent epitaxial relations in the ε-Fe_2_O_3_/GaN system. The FFT analysis confirms that the star-like reflection shape is related to the twin and antiphase boundaries existent inside the ε-Fe_2_O_3_ columns.

The field and temperature dependence of magnetization in ε-Fe_2_O_3_/GaN films have been studied in the present work by using vibrating sample magnetometer and in more detail by X-ray magnetic circular dichroism. The shape of magnetization loops is modeled using the Stoner-Wohlfarth approach of weakly interacting magnetic columns. By performing conventional M(H), full cycle M(T) and small field derivative dM/dH(T) measurements it has been demonstrated that the coercivity in ε-Fe_2_O_3_/GaN films depends drastically on the film thickness. A mild magnetic softening has been revealed to occur between 100 and 150 K similar to the one reported previously solely for epsilon ferrite nanoparticles. For the first time neutron diffraction was measured in epitaxial ε-Fe_2_O_3_ film revealing a drastic difference with previous neutron diffraction studies performed in nanoparticles and showing the presence of a distinct intensity change at 150 K. The iron coordination sensitive X-ray absorption techniques in conjunction with electron and X-ray diffraction have been used to demonstrate that the transition layer between GaN and Fe_2_O_3_ tends to have orthorhombic epsilon-like structure in a wide range of growth conditions even not optimized for ε-Fe_2_O_3_ growth. The magnetic circular dichroism measurements show that the four iron sublattices in ε-Fe_2_O_3_ response synchronously to magnetic field although with different strength and sign, at least in the surface area probed by TEY. The obtained results are supposed to shed more light onto the growth mechanisms driving the nucleation and formation of the epsilon ferrite films on GaN filling the gap between the studies of nanoparticles and epitaxial films of this exotic iron oxide polymorph.
